# Biliverdin reductase B impairs cholangiocarcinoma cell motility by inhibiting the Notch/Snail signaling pathway

**DOI:** 10.7150/jca.70323

**Published:** 2022-04-04

**Authors:** Zhihui Gao, Xiaojian Ni, Bohao Zheng, Wentao Sun, Wenze Wan, Han Liu, Xiaoling Ni, Tao Suo, Na Li, Houbao Liu, Sheng Shen

**Affiliations:** 1 Department of Nuclear Medicine, Zhongshan Hospital, Fudan University, 180 Fenglin Road, Shanghai 200032, China.; 2 Department of General Surgery, Zhongshan Hospital, Fudan University, 180 Fenglin Road, Shanghai 200032, China.; 3 Biliary Tract Disease Center of Zhongshan Hospital, Fudan University, 180 Fenglin Road, Shanghai 200032, China.; 4 Cancer Center, Zhongshan Hospital, Fudan University, 180 Fenglin Road, Shanghai 200032, China.; 5 Biliary Tract Disease Institute, Fudan University, Shanghai 200032, China.; 6 Shanghai Biliary Tract Minimal Invasive Surgery and Materials Engineering Research Center, Shanghai 200032, China.; 7 Basic Medical Institute; Key Laboratory of Cell Differentiation and Apoptosis of Chinese Ministry of Education, Shanghai Jiao Tong University School of Medicine, 280 South Chongqing Road, Shanghai 200025, China.; 8 Fudan University Shanghai Cancer Center & Institutes of Biomedical Sciences; Shanghai Medical College, Fudan University, Shanghai 200032, China.; 9 Department of General Surgery, Shanghai Xuhui Central Hospital, Zhongshan-Xuhui Hospital, Fudan University, Shanghai 200031, China.

**Keywords:** Cholangiocarcinoma, BLVRB, EMT, Snail, Notch

## Abstract

Cholangiocarcinoma (CCA) is one of the most lethal types of solid tumors worldwide. Lymph node metastasis is common in the early stage, which is associated with recurrence and reduced survival time after CCA resection. The molecular pathogenesis of CCA is complex and requires extensive investigation. It involves multiple genomic alterations and the dysregulation of signaling pathways. Biliverdin reductase B (BLVRB) is a non-redundant NAD(P)H-dependent biliverdin reductase that regulates cellular redox status by reducing biliverdin to bilirubin. This study aimed at describing the biological functions and molecular mechanisms of BLVRB in human CCA. Prognostic clinical data showed that low expression BLVRB was associated with poor prognosis and lymph node metastasis. *BLVRB* depletion accelerated epithelial-mesenchymal transition (EMT), cell migration and invasion. In contrast, BLVRB overexpression was associated with reduced EMT and cell migration and invasion in CCA. *BLVRB* suppression activated Notch signaling, and activated c-Notch enhanced EMT by upregulating Snail expression levels, thereby increasing cell migration and invasion in CCA. Our results identified an unexpected function of BLVRB in CCA migration and invasion through the regulation of Notch/Snail signaling.

## Introduction

Cholangiocarcinoma (CCA) is an aggressive cancer of the biliary tree that is associated with dismal clinical outcomes. It is the most common biliary tract malignancy and the second most common primary hepatic malignancy 1. Surgery is the only potentially curative therapeutic option, though several factors, including lymph node metastasis and vascular invasion, promote recurrence and reduce survival time after CCA resection 2-4. The high heterogeneity of CCA at the genomic, epigenetic, and molecular levels limits the efficacy of available therapies. An integrated and in-depth description of the molecular mechanisms in CCA progression could aid the development of precision therapies in advanced CCA 5, 6. The molecular pathogenesis of CCA is complex and involves multiple genomic alterations and dysregulation of signaling pathways, which require extensive investigation 7. Biliverdin reductase IXα (BLVRA) and biliverdin reductase IXβ (BLVRB) are two non-redundant NAD(P)H-dependent biliverdin reductases (BLVRs) that regulate the cellular redox cycle by reducing biliverdin (BV) to bilirubin (BR) 8, 9 . BLVRA retains specificity for the predominant BV IXα and generates BR IXα in adults. BLVRB is a comparatively promiscuous catalyst involved in the reduction of BR IXβ, the major heme catabolite during early fetal development, and other types of non-IXα BVs, several flavins, and pyrroloquinoline quinones (PQQs) 10-13. BLVRB is present in multiple organisms and is crucial for normal organ, hematopoietic, and erythrocyte/platelet functioning 12, 14, 15. BLVRB is also expressed in multiple tumors. In hepatocellular carcinoma (HCC), the restoration of BLVRB impairs the suppression of HCC growth by* miR-127-5p* 16. Unlike most enzyme families that show a high degree of active site conservation, the BLVRB family exhibits divergence, the role of which is unknown 17, 18. Preclinical studies have suggested that CCA originates from transformed cholangiocytes and hepatic progenitor cells. Clinical studies have neglected the role of BLVRB in tumorigenesis. Therefore, the present study aimed at describing the function of BLVRB at the molecular and tissue levels in CCA.

Accumulating evidence has indicated that the Notch cascade, a highly conserved pathway in most multicellular organisms, is a crucial signaling cascade in many cancer types, and aberrant Notch signaling has been implicated in CCA 19, 20. In cancer, amplification, chromosomal translocation, or mutation involving the *Notch* gene can lead to increased Notch signaling. In healthy tissues, the Notch cascade transmits information from the outer to the inner part of the cell, integrating this information into appropriate developmental or physiological responses, such as those involving Wnt/β-catenin signaling and transforming growth factor-β 21. However, Notch signaling is the only signaling pathway that converts the signal to a transcriptional response through cell-cell communication via a ligand-receptor interaction. There are four known transmembrane Notch receptors in mammals, namely Notch1, -2, -3, and -4. These receptors, together with two types of ligands (i.e., Serrate/Jagged, Jag-1 and -2; delta-like, Dll-1, -3, and -4), constitute the Notch system. Following the interaction between the ligand and its receptor, the metalloproteases, a disintegrin and metalloproteinase (ADAM) metallopeptidase domain 10 and 17 (ADAM10 and 17) cleave the Notch protein just outside the membrane. This releases the Notch extracellular domain (NECD), which continues to interact with the ligand 22. The ligand, together with the NECD, is then endocytosed by the ligand-expressing cell and cleaved by the γ-secretase complex, which releases the Notch intracellular domain (NICD). Subsequently, the NICD is translocated to the nucleus where it displaces the associated corepressors and recruits coactivators (i.e., mastermind-like family of proteins, MAML-1, -2, and -3) in association with the recombinant signal binding protein for immunoglobulin kappa J region (RBPJ) transcription factor. This molecular process leads to the expression of Notch target genes, of which the best characterized genes belong to the hairy/enhancer of split (HES) and hairy/enhancer of split-related with YRPW motif families 23.

In this study, we investigated the biological functions and molecular mechanisms of BLVRB in human CCA. We found that BLVRB was downregulated in CCA. Genetic studies have shown that the knockdown of BLVRB-mediated Notch signaling affects tumor cell migration and invasion in CCA. In addition, clinical research shows a strong association between low BLVRB expression levels and a poor prognosis in CCA. We conclude that BLVRB suppression promotes Notch activity, which is important for tumor cell dissemination. BLVRB represents a potential therapeutic target for this rare disease.

## Materials and methods

### Patients, tumor specimens, and cell lines

Clinical samples from 188 CCA patients who underwent radical surgery at Zhongshan Hospital (Shanghai, China) between 2012 and 2015 were analyzed in this study. Patients were included in the study according to the following criteria: (1) CCA was diagnosed by the department of pathology, (2) the patient did not receive preoperative chemotherapy or radiotherapy, and (3) the patient underwent radical surgery. Patient data were excluded if (1) the clinical records were incomplete or (2) follow-up information was missing. The tumor specimens were identified by biliary specialists and resected by pathologists. Patient characteristics and clinical parameters, including sex, age, tumor size, tumor number, lymph node metastasis status, liver cirrhosis, and tumor differentiation status were obtained from medical records and re-estimated by two pathologists and surgeons. The use of human tissue samples and clinical data was approved by the Ethics Committee of Fudan University (approval number: B2017-151R). All patients involved provided their informed consent. The CCA cell lines, RBE and HCCC9810, were purchased from the Shanghai Branch Cell Bank of the Chinese Academy of Sciences (Shanghai, China). Human 293T, RBE, and HCCC9810 cells were cultured in RPMI-1640 (Cat# L210KJ; BasalMedia, Shanghai, China). All media were supplemented with 10% fetal bovine serum (FBS; Cat# 10270-106; Gibco, Waltham, MA, USA) and cells were cultured under a humidified atmosphere with 5% CO_2_ at 37 °C.

### Tissue microarray analysis and immunohistochemical staining

All biopsy samples taken from the core of CCA tumoral tissues and peritumoral normal tissues were formalin-fixed, paraffin-embedded, and placed on glass slides. The slides were baked, deparaffinized, rehydrated, and endogenous peroxidase activity was blocked. Next, 5% bovine serum albumin was used to block nonspecific antigens. After antigen retrieval, the tissue sections were incubated overnight at 4 °C with anti-BLVRB (1:1,000; Lot# HPA041698; Atlas Antibodies, Bromma, Sweden), anti-cleaved Notch1 (1:1,000; Lot# 4147; Cell Signaling Technology), or anti-Snail (1:100; Lot# A11794; Abclonal) antibodies. After washing with phosphate-buffered saline, the slides were incubated with an anti-rabbit IgG antibody conjugated with horseradish peroxidase. Finally, the sections were stained with hematoxylin, dehydrated, and covered with coverslips. To evaluate the immunohistochemistry (IHC) data, we used a composite expression score ranging from 0 to 12 that was calculated as the product of the percentage of stained cells and the intensity of staining. The percentage of staining was graded as: <5% (score 0), 5-25% (score 1), 26-50% (score 2), 51-75% (score 3), and 76-100% (score 4). The intensity of staining was graded as negative (score 0), weak (score 1), moderate (score 2), or strong (score 3). Multiplication of the two scores was regarded as the final staining score 24. X-tile software was used to determine the cut-off value for the IHC scores 25, 26, according to which the patients were stratified into high- and low-score groups.

### Plasmids

To express BLVRB proteins, the corresponding open reading frame cDNA was cloned into a lentiviral recombinant pCDH-puro vector. The shRNAs used in the present study included *BLVRB* shRNA1 (GCGGTGCAAGCAGGTTACGAA) and *BLVRB* shRNA2 (GAAGGCTCATGGTGTGGACAA), which were cloned into a pLKO.1-puro vector. Lentiviruses were generated by cotransfection of one of the above recombinant plasmids with packaging plasmids (psPAX2 and pMD2G) into 293T cells using polyethyleneimine (23966; Polysciences, Warrington, PA, USA). To establish stable cells, the transfected cells were treated with 4 mg/mL puromycin for 48 h after lentiviral infection.

### Western blotting

Cells were lysed on ice for 20 min in radioimmunoprecipitation assay lysis buffer (150 mM NaCl, 0.1% SDS, 50 mM Tris-HCl [pH 7.5], 1% NP-40, and 1% sodium deoxycholate) supplemented with a protease inhibitor cocktail (Cat# p8340; Sigma-Aldrich) and a phosphatase inhibitor cocktail (Cat# P2850, Sigma-Aldrich). The protein concentration of each sample was measured using a bicinchoninic acid assay kit (Cat# BCA02; Dingguo, Beijing, China). The proteins were subsequently separated by polyacrylamide gel electrophoresis and transferred to polyvinylidene fluoride membranes, which were incubated with a primary antibody and then with a secondary antibody. The blots were visualized using an ImageQuant LAS 4000 (GE Healthcare, Chicago, IL, USA) with an enhanced chemiluminescence assay. The following antibodies were used: anti-BLVRB (1:1,000; Lot# HPA041698; Atlas Antibodies), anti-BLVRA (1:1,000; Lot# HPA042865; Atlas Antibodies), anti-E-cadherin (1:1,000; 2Q663; Lot# A0313; Santa Cruz Biotechnology), anti-Claudin-1 (1:1,000; D5H1D; Lot# 13255T; Cell Signaling Technology), anti-N-cadherin (1:1,000; D4R1H; Lot# 13116; Cell Signaling Technology), anti-Snail (1:1,000; C15D3; Lot# 3879; Cell Signaling Technology), anti-ZEB1 (1:1,000; D80D3; Lot# 3396; Cell Signaling Technology), anti-cleaved Notch1 (1:1,000; Val1744; D3B8; Lot# 4147; Cell Signaling Technology), anti-non-phospho (active) β-catenin (1:1,000; Ser33/37/Thr41; Lot# 4270; Cell Signaling Technology), anti-non-phospho (active) β-catenin (1:1,000; D10A8; Lot# 8480; Cell Signaling Technology), anti-β-actin (1:2,000; C4; Lot# sc-47778; Santa Cruz Biotechnology), and horseradish peroxidase-conjugated secondary antibody (1:2,000; Cat# SA00001-1 or SA00001-2; Proteintech).

### Real-time PCR

Total RNA was isolated using TRIzol reagent (Cat# 15596026, Invitrogen, Carlsbad, CA, USA) and cDNA was synthesized using a PrimeScript RT Reagent Kit (Cat# RR037A, Takara, Kyoto, Japan), with a mixture of oligo dT and random primers, after genomic DNA elimination. mRNA expression levels were measured by real-time PCR using an ABI-7500 system (Applied Biosystems, Waltham, MA, USA) and a 2×SYBR Green qPCR Master kit (Cat# A0001, EZBioscience, Roseville, MN, USA) according to the manufacturer's instructions. Relative expression levels were calculated by determining the threshold cycle values of the samples. All data were normalized to β-actin as an internal control.

The primers used for real-time PCR analysis were as follows: *BLVRB* forward, 5ʹ- CTCATGGTGTGGACAAGGTCGT-3ʹ and reverse, 5ʹ-CATCACAGCCACGTACTTCAGG-3ʹ; *β-actin* forward, 5ʹ-GCGGGAAATCGTGCGTGACATT-3ʹ and reverse, 5ʹ-GATGGAGTTGAAGGTAGTTTCG-3ʹ; *E-cadherin* forward, 5ʹ-CGAGAGCTACACGTTCACGG-3ʹ and reverse, 5ʹ-GGGTGTCGAGGGAAAAATAGG-3ʹ; *N-cadherin* forward, 5ʹ-TGCGGTACAGTGTAACTGGG-3ʹ and reverse, 5ʹ-GAAACCGGGCTATCTGCTCG-3ʹ; *Snail* forward, 5ʹ-TCGGAAGCCTAACTACAGCGA-3ʹ and reverse, 5ʹ-AGATGAGCATTGGCAGCGAG-3ʹ; and *Slug* forward, 5ʹ-TGTGACAAGGAATATGTGAGCC-3ʹ and reverse, 5ʹ-TGAGCCCTCAGATTTGACCTG-3ʹ.

### RNA-seq

After total RNA was extracted, mRNA was isolated using oligo (dT) magnetic beads and cut into small fragments for cDNA synthesis. Libraries were generated using a NEBNext Ultra RNA Library Prep Kit (New England Biolabs, Ipswich, MA, USA) for an Illumina system, following the manufacturer's instructions. Sequencing was performed using a HiSeq XTEN platform (Illumina, San Diego, CA, USA).

### Transwell assays

Transwell assays of tumor cells were performed in 24-well transwell plates with an 8-μm pore size, according to the manufacturer's instructions (Corning, NY, USA). For tumor cell migration/invasion assays, cells (3 × 10^4^) were suspended in 200 μL of serum-free Dulbecco's modified Eagle's medium (DMEM) and seeded on the upper chamber. The lower chamber was filled with DMEM supplemented with 10% FBS. After incubation at 37 °C for 20 h, the cells that migrated to the lower chamber were fixed with methanol for 15 min at room temperature and stained with 0.1% crystal violet for another 15 min. The medium surface in the upper chamber was carefully wiped with a cotton-tipped applicator. Cells that migrated to the lower chamber were counted in five non-overlapping fields and photographed. For invasion assays, the bottom chamber was coated with Matrigel basement membrane matrix (BD Biosciences, San Jose, CA, USA).

### Luciferase reporter assays

To assess the effect of BLVRB on Notch-mediated transcriptional activity, we used a traditional dual-luciferase assay consisting of four *Notch*-sensing* CBF1*-binding site reporters (normalized to a control promoter driving *Renilla* luciferase), as previously described 27. pCBFRE-luc was donated by Nicholas Gaiano (plasmid# 26897; Addgene, Watertown, MA, USA). Briefly, cells were co-transfected with the *CBF1*-luciferase reporter construct and a *Renilla* luciferase reporter plasmid. Luciferase activity was examined 24 h after transfection using a dual-luciferase reporter assay system (Promega, Madison, WI, USA) according to the manufacturer's instructions. Firefly luciferase activity from the *CBF1* reporter construct was normalized to the control *Renilla* luciferase included in the kit. Luciferase activity was expressed as the fold increase relative to unstimulated conditions.

### Statistical analysis

Statistical analyses were performed using SPSS 13.0 statistical software (IBM SPSS, Chicago, IL, USA). A chi-squared or rank-sum test was used to investigate the relationship between BLVRB expression and clinicopathological factors. A two-tailed Student's *t*-test or analysis of variance (ANOVA) was used to assess the differences between the control and treatment groups. Survival curves were plotted using the Kaplan-Meier method and compared with a log-rank test. All assays were repeated at least three times. The differences were considered to be statistically significant at *P* < 0.05.

## Results

### Decreased BLVRB levels predicted a poor prognosis in cholangiocarcinoma

**Figure 1:**
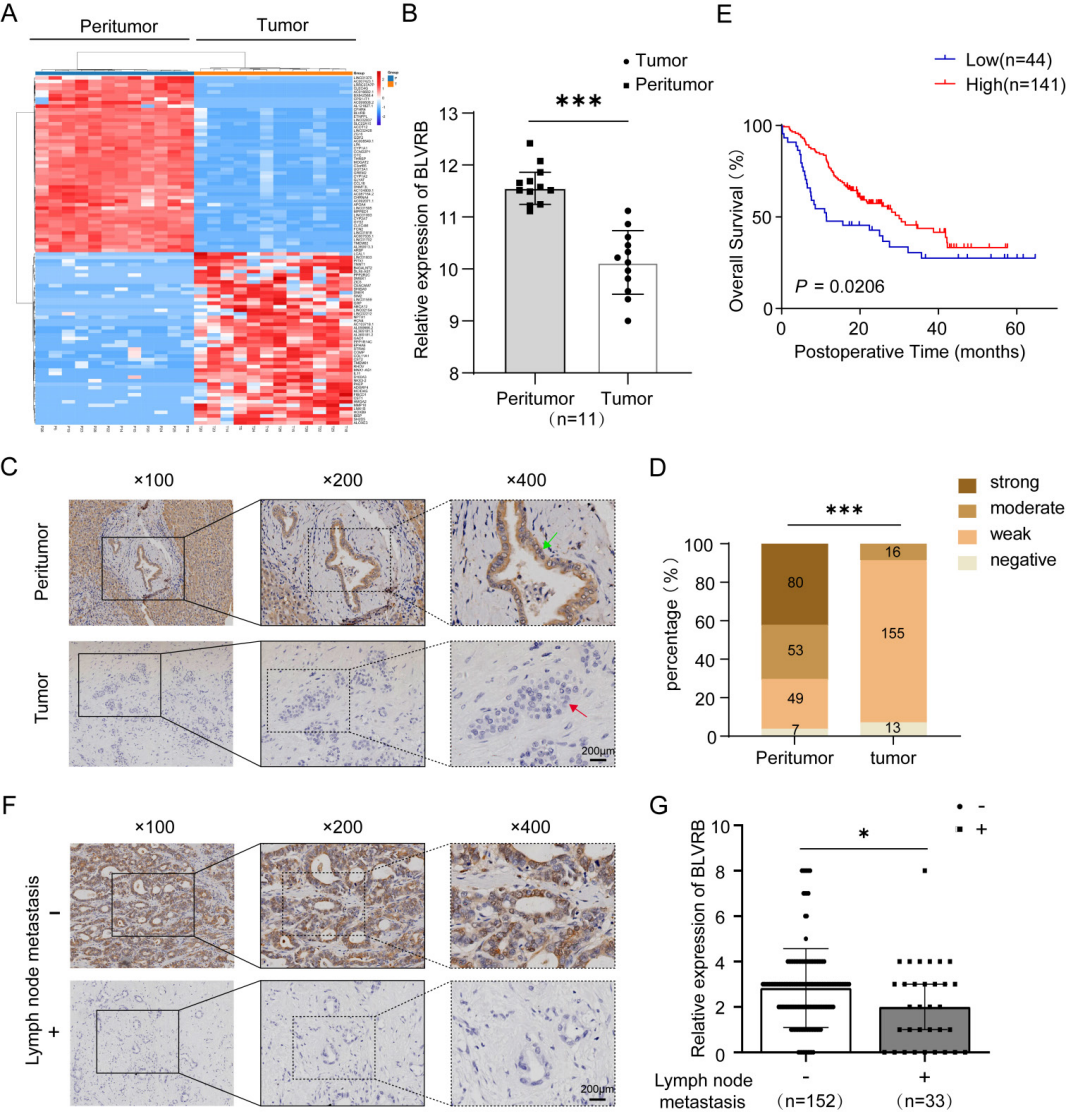
** The expression pattern of BLVRB in cholangiocarcinoma. A.** Hierarchical clustering of differentially expressed mRNAs from 11 paired tissue samples from cholangiocarcinoma (CCA) patients. **B.** The mRNA expression level of *BLVRB* in 11 paired tissue samples. *BLVRB* levels were lower in tumor tissues than in peritumoral normal tissues. ***P < 0.001. **C.** Negative staining of BLVRB in CCA (lower, red arrows) and positive staining of BLVRB in matched peritumor tissue (upper, green arrows). Scale bar: 200 µm. **D.** The BLVRB score was lower in tumor tissues than in peritumoral normal tissues. ***P < 0.001. **E.** The Kaplan-Meier analysis showed that OS rates were significantly higher in patients with high levels of BLVRB expression than in patients with low levels of BLVRB expression (*P* = 0.021). **F.** Representative immunostaining images of BLVRB in CCA without lymph node metastasis and with lymph node metastasis. Scale bar: 200 µm. **G.** The BLVRB score in tumor tissues of patients without lymph node metastasis and with lymph node metastasis. The BLVRB scores were lower in patients with lymph node metastasis than in patients without lymph node metastasis.

We compared the transcriptomes of 11 paired tumor and peritumor tissues and found differentially expressed genes in CCA. *BLVRB* levels were lower in tumor tissues than in peritumor tissues (Fig. 1A). According to the RT-PCR analysis, *BLVRB* was markedly lower in tumor tissues compared to peritumor tissues (Fig. 1B, *P* < 0.001). According to IHC staining, BLVRB was localized in the cytoplasm and nucleus of bile duct tissues. In normal peritumor biliary tissues, we observed a strong expression level in 42.3% (80/189), moderate expression level in 28.0% (53/189), weak expression level in 25.9% (49/189), and negative expression in 3.7% (7/189) (Fig. 1C, 1D and S1, *P* < 0.001). The expression level of BLVRB in CCA tissues was significantly lower than that in normal peritumor biliary tissues. None of the CCA tissue samples showed a strong BLVRB expression level, 8.7% (16/184) showed a moderate expression level, 84.2% (155/184) showed a weak expression level, and 7.1% (13/184) of samples were negative for BLVRB expression (Fig. 1C and 1D, *P* < 0.001). To illustrate the clinical relevance of BLVRB expression in CCA, patients were grouped according to the expression level of BLVRB (high: final staining score ≥ 2; low: final staining score < 2). According to the Kaplan-Meier analysis, the overall survival (OS) rate of the group with low BLVRB expression levels was lower than that of the group with high BLVRB expression levels (Fig. 1E, *P* = 0.021). This suggests that the downregulation of BLVRB is associated with poor prognosis in patients with CCA. A low BLVRB expression level was strongly associated with lymph node metastasis (Table 1, *P* = 0.016). No association was observed between BLVRB expression level and gender, age, tumor size, tumor number, vascular invasion, differentiation, or liver cirrhosis. Next, we divided patients with CCA into two groups according to lymph node metastasis and assessed the BLVRB expression pattern by IHC. The IHC scores were lower in patients with lymph node metastasis than in patients without (Fig. 1F and 1G, *P* < 0.05). Therefore, BLVRB levels seem to be negatively associated with lymph node metastasis.

### *BLVRB* depletion enhanced cell migration and invasion in cholangiocarcinoma *in vitro*

**Figure 2:**
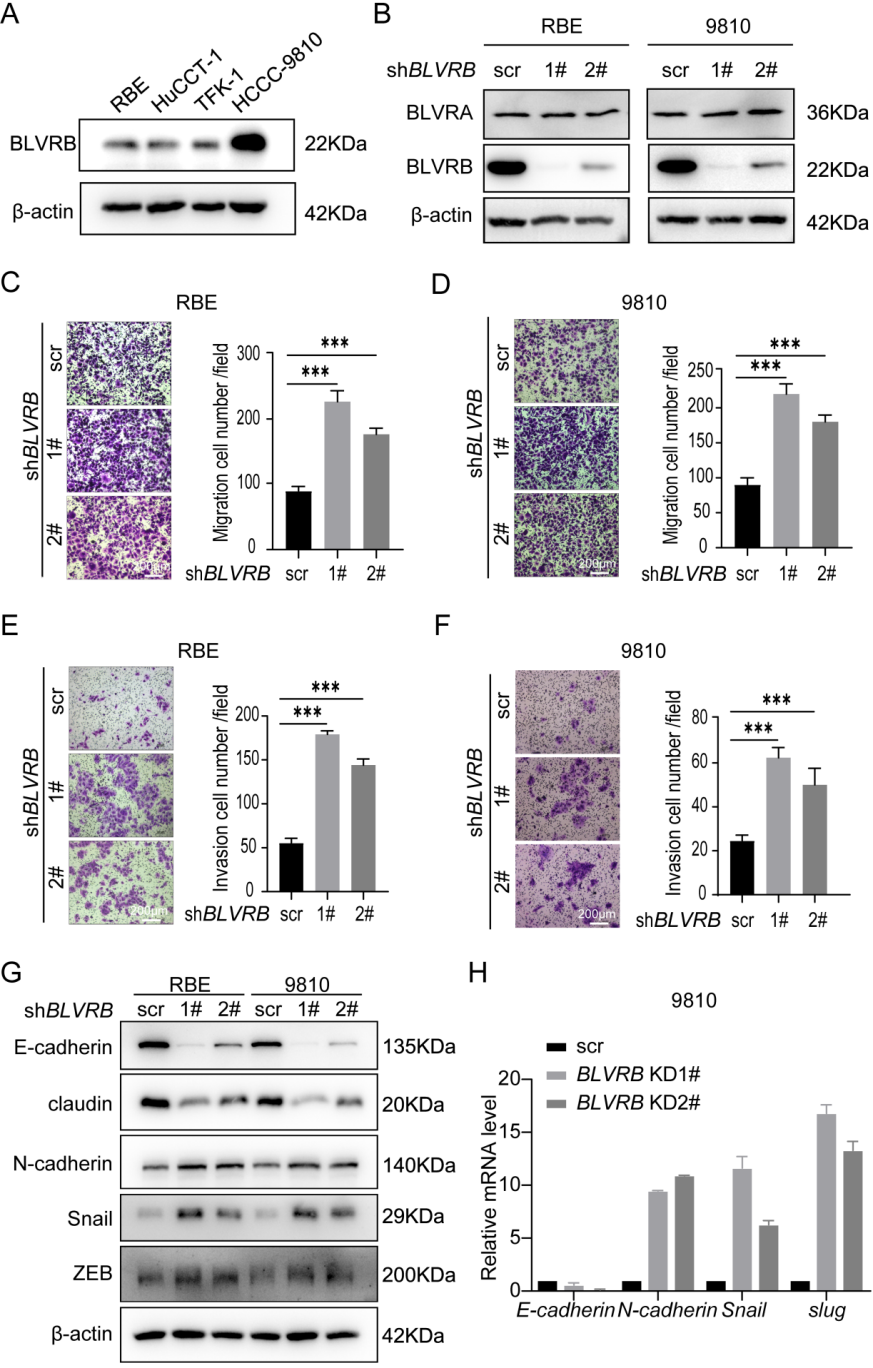
** Effect of BLVRB depletion on cholangiocarcinoma cell migration and invasion *in vitro.* A.** Relative BLVRB protein levels in cholangiocarcinoma cell lines. **B.** Protein levels of BLVRB and BLVRA were verified by western blotting analysis after stable knockdown of *BLVRB*. **C and D.** RBE and 9810 cell migration from the upper transwell chamber to the lower chambers. BLVRB knockdown promoted the migration of RBE and 9810 cells. ***P < 0.001. Scale bar: 200 µm. **E and F.** RBE and 9810 cell invasion from the upper transwell chamber to the lower chamber. *BLVRB* knockdown promoted the invasive ability of RBE and 9810 cell lines. ***P < 0.001. Scale bar: 200 µm. **G.** Protein levels of key epithelial-mesenchymal transition (EMT) markers, including E-cadherin, claudin, N-cadherin, Snail, and ZEB, after *BLVRB* knockdown. **H.** The mRNA levels of key EMT markers, including *E-cadherin*, *N-cadherin*, *Snail*, and *Slug*, after *BLVRB* knockdown. All experiments were performed in triplicate.

**Figure 3:**
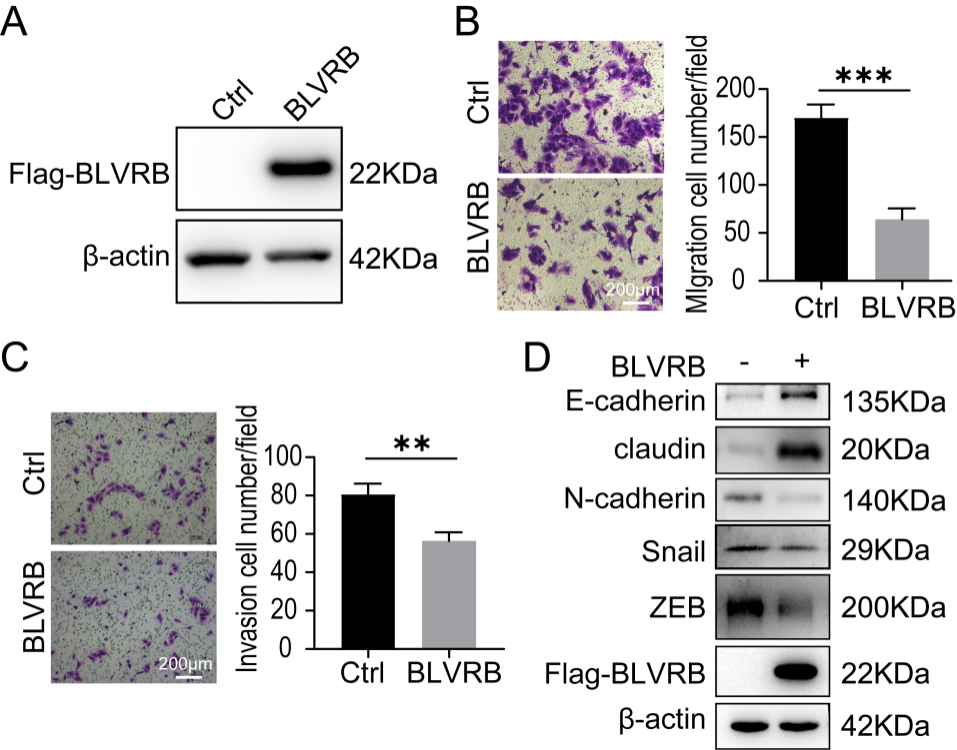
** Effect of BLVRB overexpression on cholangiocarcinoma cell migration and invasion *in vitro* A.** Stable *BLVRB* overexpression in RBE cells was verified by western blotting analysis. **B.** RBE cell migration from the upper transwell chamber to the lower chamber. The overexpression of *BLVRB* impaired the migration of RBE cells. **C.** RBE cell invasion from the upper transwell chamber to the lower chamber. The overexpression of *BLVRB* impaired the invasive ability of RBE cells. **D.** Protein levels of key EMT markers, including E-cadherin, claudin, N-cadherin, Snail, and ZEB, after the overexpression of *BLVRB* in RBE cells. All experiments were performed in triplicate.

To evaluate the impact of BLVRB on CCA metastasis at the cellular level, we first determined endogenous BLVRB levels in different CCA cell lines. BLVRB expression levels were higher in HCCC9810 cells than in HuCCT-1, TFK-1, and RBE cells (Fig. 2A). We silenced *BLVRB* in HCCC9810 and RBE cells, and the efficiency of *BLVRB* knockdown was verified by western blotting (Fig. 2B). *BLVRB* knockdown had no effect on BLVRA (Fig. 2B). The migration ability of both RBE and 9810 cells increased after *BLVRB* knockdown, as determined by transwell migration assays (Fig. 2C and 2D). The invasion ability of RBE and 9810 cells was enhanced after *BLVRB* knockdown, as evidenced by the transwell Matrigel assay results (Fig. 2E and 2F). EMT is closely related to enhanced cell motility, and it enables the development of invasive properties and metastatic growth characteristics in tumor cells. We assessed the effect of *BLVRB* depletion on EMT in RBE and 9810 cells. *BLVRB* knockdown induced upregulation of the mesenchymal marker N-cadherin and downregulation of epithelial markers E-cadherin and claudin. Moreover, Snail and ZEB, which are the transcription repressors of E-cadherin, were also upregulated after *BLVRB* knockdown (Fig. 2G). Knockdown of *BLVRB* consistently decreased the mRNA level of *E-cadherin* and enhanced the mRNA levels of *N-cadherin*, *Snail*, and *Slug*, according to the RT-PCR results (Fig. 3H). These results suggest that BLVRB depletion participates in an EMT-like switch that facilitates cell migration and invasion in CCA.

### BLVRB overexpression reduced cell migration and invasion in cholangiocarcinoma *in vitro*

HuCCT-1, TFK-1, and RBE cells had similarly low BLVRB expression levels (Fig. 2A). We overexpressed BLVRB in RBE cells and then selected and identified stably transfected cells with puromycin, as shown in Fig. 3A. The overexpression of BLVRB markedly blocked RBE cell migration and invasion (Fig. 3B and 3C). EMT-related markers were detected after BLVRB overexpression. As shown in Fig. 3D, *BLVRB* overexpression decreased the protein levels of the mesenchymal markers, N-cadherin, Snail, and ZEB, but increased the expression levels of the epithelial markers, E-cadherin and claudin. Overall, these results suggest that BLVRB plays a role in the suppression of cell migration and invasion.

### BLVRB effectively suppressed the activation of the Notch signaling pathway

**Figure 4:**
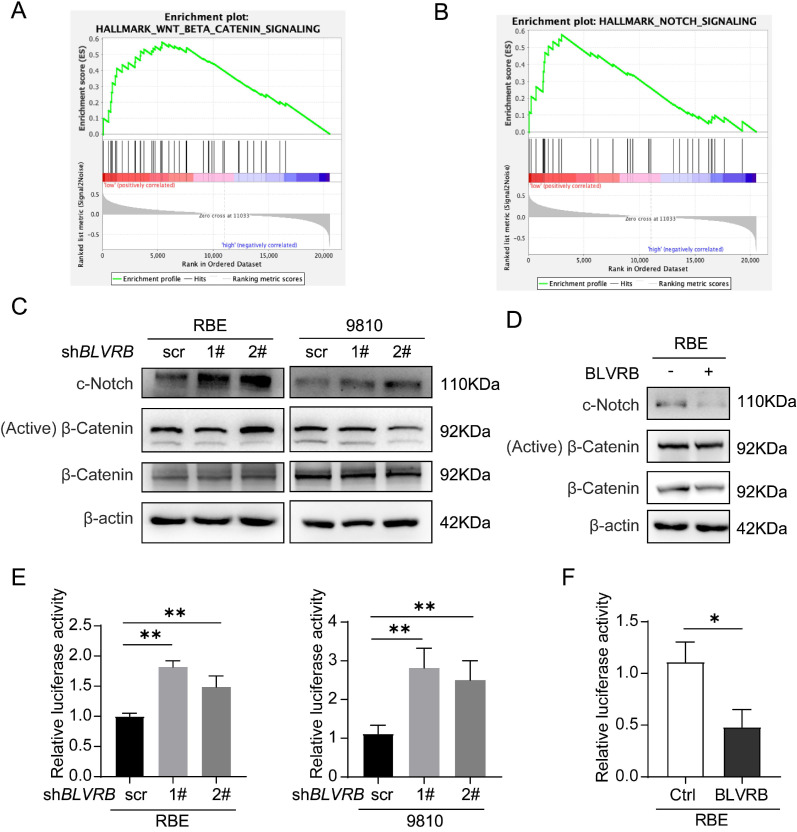
** BLVRB effectively suppressed the activation of the Notch signaling pathway. A and B.** Gene set enrichment analysis indicated that a low expression level of *BLVRB* was positively associated with the Notch and Wnt/β-catenin signaling pathways. **C.** Protein levels of c-Notch, active β-catenin, and total β-catenin after *BLVRB* knockdown. **D.** Protein levels of c-Notch, active β-catenin, and total β-catenin after *BLVRB* overexpression. **E and F.** Dual-luciferase assay shows Notch activity after knockdown or overexpression of *BLVRB*. The experiments were performed in triplicate.

To explore the mechanism by which BLVRB regulates CCA development, we identified genes whose expression patterns correlated with those of BLVRB in the Cancer Genome Atlas CCA dataset and performed gene set enrichment analysis (GSEA). Low *BLVRB* expression levels were positively associated with the Notch and Wnt/β-catenin signaling pathways (Fig. 4A and 4 B). Western blotting analysis showed that *BLVRB* knockdown increased cleaved Notch1 protein levels, whereas overexpression had the opposite effect (Fig. 4C and 4D). However, *BLVRB* knockdown or overexpression had no effect on activated β-catenin protein levels (Fig. 4C and 4D). These results suggest that BLVRB is involved in regulating the Notch signaling pathway, but not the Wnt/β-catenin signaling pathway. According to the dual-luciferase reporter assay, knockdown of *BLVRB* in RBE and 9810 cells significantly increased Notch-mediated transcriptional activity, whereas overexpression of BLVRB inhibited the Notch-mediated transcriptional response (Fig. 4E and 4F). These results suggest that BLVRB participates in the EMT switch to affect cell motility through the Notch signaling pathway. The Notch pathway was further evaluated for its role in the BLVRB-mediated inhibition of metastasis.

### BLVRB depletion promoted cell migration by activating the Notch/Snail signaling pathway

**Figure 5:**
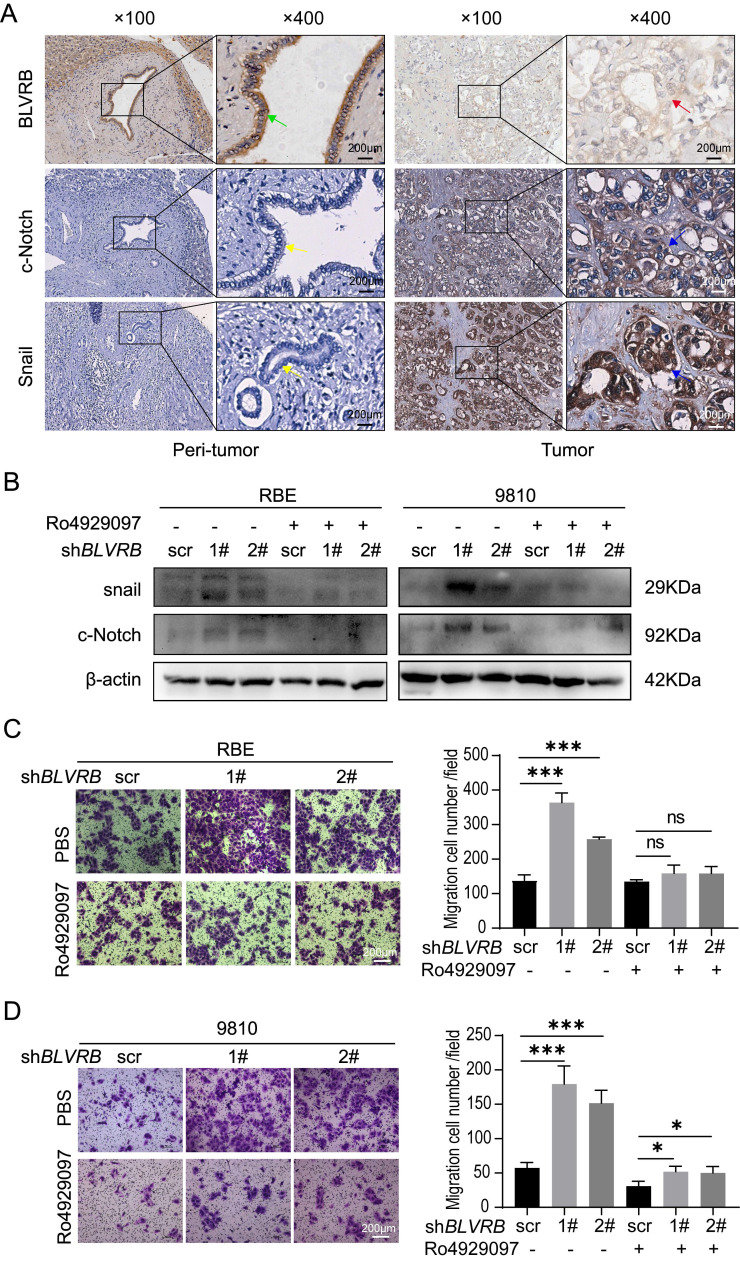
** BLVRB depletion promoted cell migration by activating the Notch/Snail signaling pathway. A.** The association of BLVRB with c-Notch and Snail in CCA tissues was assessed using IHC assays. Weak staining of BLVRB (red arrows) and positive staining of c-Notch and Snail (blue arrows) in CCA are shown in the right-hand column; positive staining of BLVRB (green arrows) and negative staining of c-Notch and Snail (yellow arrows) in matched peritumor tissue are shown in the left-hand column. Scale bar: 200 µm. **B.** Protein levels of c-Notch and Snail after *BLVRB* depletion. **C.** The effect of Ro4929097 on RBE cell migration after *BLVRB* depletion. **D.** The effect of Ro4929097 on 9810 cell migration after *BLVRB* depletion.

*Snail* is a typical downstream Notch target gene 28. To further elucidate the effect of BLVRB on Notch signaling-related transcriptional activity, we performed an IHC assay and found that the BLVRB protein was positively associated with c-Notch and Snail (Fig. 5A). Next, we measured Snail protein levels after treatment with the γ-secretase inhibitor, Ro4929097, which specifically inhibits the activation of Notch signaling. Inhibition of the Notch signaling pathway by Ro4929097 treatment reduced Snail expression levels and blocked the effect of *BLVRB* depletion on Snail expression (Fig. 5B). Thus, *BLVRB* depletion seems to regulate EMT in CCA through the Notch/Snail pathway. To determine whether BLVRB regulated the migration and invasion of CCA cells through the Notch/Snail pathway, we assessed the effect of Ro4929097 on sh*BLVRB*-induced CCA cell migration. The transwell assay results showed that *BLVRB* depletion increased the number of migrated cells, while Ro4929097 treatment decreased cell migration, even in *BLVRB*-depleted cells (Fig. 5C and 5D). These results suggest that *BLVRB* silencing promotes CCA cell migration by upregulating the activation of the Notch/Snail pathway.

## Discussion

This is the first study to explore the function of BLVRB in CCA metastasis. We found that BLVRB, an isoenzyme of BLVRA, was downregulated in CCA and that low BLVRB expression levels in clinical specimens were associated with poor prognosis. In *in vitro* assays, lower expression levels of BLVRB were important for the EMT, migration, and invasion of CCA cells. Our results revealed an unexpected function of BLVRB in CCA migration and invasion via Notch signaling.

CCA shows a high degree of genetic heterogeneity, and its prognosis remains dismal. The average 5-year OS rate is ~10-30%, and the median OS time across all stages of progression is ~10-28 months 29-31. Lymph node metastasis occurs in 75% of patients with early-stage CCA (T1 and T2) and often implies a poor prognosis 32, 33. Malignant cells that favor metastasis acquire key phenotypic advantages, such as increased cell invasiveness and migration. EMT plays an important role in tumor metastasis and it leads to functional changes in cell migration and invasion. Radical resection is currently one of the most effective treatment methods, though ~40% of patients die of recurrence within 3 years after surgery. Precision medicine is required to improve patient outcomes.

Heme is one of the most abundant molecules in the body and its core function is the synthesis of hemoglobin/myoglobin, which is involved in the transport of O_2_/CO_2_ in blood and tissue, as well as redox enzymes and cytochromes in mitochondria. The tissue concentration of heme, BV and BR, is tightly controlled. Heme oxygenase-1 (HO-1) produces BV by heme degradation, while BLVRs generate BR by conversion of BV. Two BLVRs, BLVRB and BLVRA, are encoded in humans. These enzymes share little sequence similarity, though their structures are typical of the short-chain dehydrogenase/reductase superfamily 34. BLVRB, together with BLVRA, promotes isomeric bilirubin generation and has cellular antioxidant functions. BLVRA retains specificity for BV IXα, the predominant type BV in adults, while BLVRB is promiscuous and catalyzes the reduction of non-IXα BVs, such as IXβ, which is the predominant BV at birth. BLVRA possesses a classical protein kinase domain that is activated in response to BV binding to its enzymatic site and initiates the downstream mitogen-activated protein kinases (MAPK) and phosphatidylinositol 3-kinase (PI3K) pathways. This links BLVRA activity to cell growth and survival pathways 35. BR acts as an antioxidant and BLVRA deficiency increases the susceptibility of mice to oxidative stress-induced hepatic steatosis in the absence of insulin resistance 36, 37.

Compared to BLVRA, few studies have considered the function of BLVRB. In the present study, BLVRB impaired CCA migration and invasion via Notch signaling. However, the physiological functions of BLVRB remain unclear. Recent evidence in humans has implicated redox-defective BLVRB in a thrombopoietic mechanism determining hematopoietic lineage fate. BLVRB is highly expressed in multiple organisms. It is vital for normal organ function, except for hematopoietic and erythrocyte/platelet functions. In this study, we found that low levels of BLVRB expression in clinical specimens were associated with poor prognosis and that BLVRB overexpression inhibited the migration and invasion of CCA cells. Moreover, the suppression of CCA cell migration and invasion by BLVRB may be dependent on catalytic activity (data not shown). Future research should attempt a more detailed characterization of BLVRB-regulated metabolites and description of the molecular events in BLVRB-regulated tumorigenesis.

In the human liver, the Notch signaling pathway is the key regulator of normal cholangiocyte differentiation 38, 39. Notch signaling plays multiple roles in development and tissue homeostasis, and these roles may be subverted during oncogenic transformation. Notch signaling occurs through direct interaction between the Notch receptor and its ligand. There are four types of receptors and two types of ligands that constitute the Notch system, though several other components transduce and regulate signals. Cumulating evidence indicates that aberrant Notch signaling is implicated in CCA. Aberrant expression of Notch1 in intrahepatic CCA (iCCA) was associated with increased tumor size, while the overexpression of Notch4 was related to poor OS 40. Notch1, -2, -3, and -4 and Hes-1 were respectively expressed in 50.0%, 56.1%, 42.4%, 6.1%, and 81.8% of extrahepatic CCA specimens. In addition, patients overexpressing at least one of the Notch1, 2, or 3 receptors exhibited a poor prognosis 20. A recent investigation revealed that most iCCA samples overexpress the Notch1 receptor 19.

The Notch signal transduction cascade results in the proteolytic processing of the Notch receptor and the subsequent assembly of a transcriptional coactivator complex containing the NICD and the transcription factor, RBPJ. RBPJ is also known as CBF1 (C promoter binding factor 1), KBF2 (H-2K binding factor-2), and RBPJκ (recombination signal binding protein for immunoglobulin kappa J region). RBPJ belongs to the CSL (*Homo sapiens* CBF1, *Drosophila melanogaster* suppressor of hairless, and *Caenorhabditis elegans* Lag-1) protein family. In the absence of a Notch signal, RBPJ remains bound to Notch target genes, where it represses transcriptional output 41. Activation of the Notch pathway leads to the release of the NICD from the cell membrane, which, upon nuclear translocation, converts RBPJ from a repressor to an activator of transcription via the recruitment of additional coactivators. In the liver, Notch signaling is essential for biliary fate and morphogenesis 42-45. Despite the wealth of data suggesting a role for Notch in CCA, little evidence exists to support a causative role for Notch in tumor initiation in human CCA.

In conclusion, the knockdown of *BLVRB* participated in the activation of the Notch pathway. As in many other cancer types, the activation of Notch signaling led to EMT induction in CCA cell lines, thus conferring tumor progression. In summary, we found that *BLVRB* depletion promoted CCA cell migration and invasion by activating Notch signaling, which is a novel function of BLVRB. However, this mechanism requires further elucidation, as it is a potential target for the prevention and treatment of CCA.

## Supplementary Material

Supplementary figure.

## Figures and Tables

**Table 1:** The relationship between clinical parameters and the expression level of BLVRB

Variable	Low (n=44)	High (n=141)	Statistic	*P* value
**Gender**				
Male	27	79	χ^2^=0.390	0.532
Female	17	62		
**Age**				
≤60	21	66	χ^2^=0.011	0.915
>60	23	75		
**Tumor size**				
≤5	19	67	χ^2^=0.253	0.615
>5	25	74		
**Tumor number**				
Single	33	103	χ^2^=0.066	0.798
Multiple	11	38		
**Vascular invasion**				
Present	44	136	χ^2^=0.539	0.463
Absent	0	5		
**Differentiation**				
Poor	2	10	Z=-1.764	0.078
Moderate~Poor	6	5		
Moderate	17	54		
Well~Moderate	6	2		
Well	13	70		
**Lymph node metastasis**				
Absent	30	120	χ^2^=6.262	**0.012**
Present	14	21		
**Cirrhosis**				
Absent	38	116	χ^2^=0.403	0.526
Present	6	25		
