# Supplementary material for: Biliverdin reductase B impairs cholangiocarcinoma cell motility by inhibiting the Notch/Snail signaling pathway
Source: J Cancer. 2022 Apr 4;13(7):2159–70. doi: 10.7150/jca.70323 (PMC9066219; doi:10.7150/jca.70323)
Supplement: Supplementary file 1 — Supplementary figure. [file jcav13p2159s1.pdf]

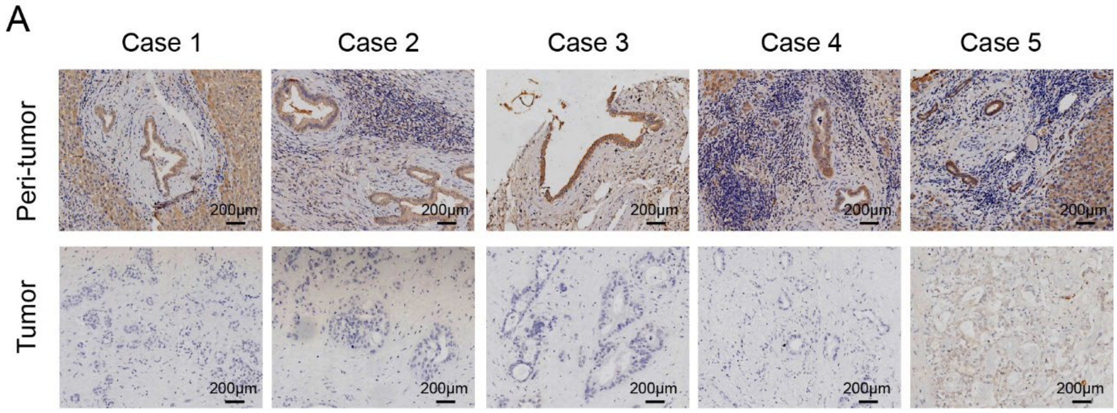

Figure S1. BLVRB depletion promoted cell migration by activating the Notch/Snail signaling pathway

A. Immunostaining images of BLVRB in 5 paired CCA and matched peritumor tissues. Scale bar: 200 μm.
